# Thyroid-specific questions on work ability showed known-groups validity among Danes with thyroid diseases

**DOI:** 10.1007/s11136-014-0896-0

**Published:** 2014-12-19

**Authors:** Mette Andersen Nexo, Torquil Watt, Steen Joop Bonnema, Laszlo Hegedüs, Åse Krogh Rasmussen, Ulla Feldt-Rasmussen, Jakob Bue Bjorner

**Affiliations:** 1 The National Research Centre for the Working Environment, Lersø Parkallé 105, 2100 Copenhagen, Denmark; 2 Section of Social Medicine, Department of Public Health, University of Copenhagen, Gothersgade 160, 1014 Copenhagen, Denmark; 3 Department of Medical Endocrinology, Copenhagen University Hospital (Rigshospitalet), Blegdamsvej 9, 2100 Copenhagen, Denmark; 4 Department of Endocrinology and Metabolism, Odense University Hospital, Sdr. Boulevard 29, 5000 Odense, Denmark; 5 QualityMetric (an Optum Company), 24 Albion Road, Building 400, Lincoln, RI 02865 USA

**Keywords:** Thyroid disease, QOL, Scale validation, Work ability, Hyperthyroidism, Hypothyroidism

## Abstract

**Purpose:**

We aimed to identify the best approach to work ability assessment in patients with thyroid disease by evaluating the factor structure, measurement equivalence, known-groups validity, and predictive validity of a broad set of work ability items.

**Methods:**

Based on the literature and interviews with thyroid patients, 24 work ability items were selected from previous questionnaires, revised, or developed anew. Items were tested among 632 patients with thyroid disease (non-toxic goiter, toxic nodular goiter, Graves’ disease (with or without orbitopathy), autoimmune hypothyroidism, and other thyroid diseases), 391 of which had participated in a study 5 years previously. Responses to select items were compared to general population data. We used confirmatory factor analyses for categorical data, logistic regression analyses and tests of differential item function, and head-to-head comparisons of relative validity in distinguishing known groups.

**Results:**

Although all work ability items loaded on a common factor, the optimal factor solution included five factors: role physical, role emotional, thyroid-specific limitations, work limitations (without disease attribution), and work performance. The scale on thyroid-specific limitations showed the most power in distinguishing clinical groups and time since diagnosis. A global single item proved useful for comparisons with the general population, and a thyroid-specific item predicted labor market exclusion within the next 5 years (OR 5.0, 95 % CI 2.7–9.1).

**Conclusions:**

Items on work limitations with attribution to thyroid disease were most effective in detecting impact on work ability and showed good predictive validity. Generic work ability items remain useful for general population comparisons.

## Introduction

Thyroid disorders are common chronic diseases [1–3] associated with increased somatic [4, 5] and psychiatric morbidity [6–8] and excess mortality [9, 10]. Traditionally, health-related quality of life (HRQOL) has not been formally assessed, since treatment has been considered effective in relieving symptoms. However, recent studies have documented reduced HRQOL in thyroid diseases [11–17] even in well-treated patients [12, 15–18]. A thyroid-specific patient HRQOL instrument, the ThyPRO, has been developed [19] following recent guidelines from the US Food and Drug Administration [20, 21].

The ability to work and support oneself is an important aspect of HRQOL. Many thyroid patients contract the disease at working age. In addition to the lack of focus on HRQOL impact, few studies have evaluated work ability [11, 22, 23], because adequately treated thyroid disease was not assumed to have a major impact on work [24]. However, recent register-based studies have demonstrated that thyroid patients have an increased risk of sick leave, diminished earnings and exclusion from the labor force [25–28]. Thus, HRQOL assessment of thyroid patients should include careful measurement of work ability. Therefore, we undertook a project to improve the assessment of work ability in the ThyPRO, which currently only use one out of 98 items to access work ability.

Work ability is a dynamic concept that concerns an individual’s capacity to perform work tasks and depends on health related, individual, and contextual factors [29]. We used the conceptual framework of the World Health Organization (WHO) “Health and Disability” model [30]. Together with individual and contextual factors, a disease can impact a body’s function or structure and impact an individual’s ability to carry out activities at work. Most thyroid diseases affect the metabolism and thus all the psychological and physiological processes in the body. Hypothyroidism has been related to severe fatigue, and hyperthyroidism has been related to psychological distress [17, 31, 32]. These mechanisms are presumably associated with the experienced work role limitations [11, 22, 23] and the difficulties maintaining employment observed in many thyroid diseases [25–28].

A wide range of self-report questionnaires has been developed to measure health-related work disability in different clinical populations [33–35]. These instruments vary greatly in their conceptualization of work ability and can be described by at least three properties:

1. *Question specificity* Some instruments, e.g., the Work Ability Index (WAI) [36], ask for a global assessment of work ability, while others, such as the Work Limitations Questionnaire (WLQ) [37], focus on specific work activities.
2. *Attribution* Some instruments (e.g., the SF-36) [38], ask about limitations due to physical or mental health factors; other questions, such as the single item on work limitations in the ThyPRO, examine limitations attributed to a specific disease, while other instruments make no attribution at all.
3. *Individual or contextual factors* Instruments like the WAI [36] contain questions that include contextual factors, such as the ability to meet the physical and mental demands of the job. Other instruments refer to individual skill level, while other instruments do not allude to individual or contextual factors.

The overall objective of this study was to identify the best approach to work ability assessment in thyroid disease. Based on the literature, review of existing questionnaires, and interviews with thyroid patients [32], we selected, revised, and developed a broad set of items on work ability, collected data from thyroid patients, and undertook analyses with four aims:

1. To evaluate the factor structure of the items, to develop one or several work ability scales based on the factor model, and to test the stability of these scales across age, gender, and thyroid diseases.
2. To identify which items best differentiate between persons with and without thyroid disease. We hypothesized that patients with thyroid diseases have worse work ability than the general population (hypothesis a).
3. To identify the work ability scales that best differentiate between different types of thyroid diseases. We assumed that work ability is impacted by diseases with hypothyroid or hyperthyroid functioning (hypothesis b) and that work ability is worse within the first year after diagnosis compared to subsequent years (hypothesis c).
4. To evaluate the predictive validity of self-assessed work ability on a single item for predicting exclusion from the labor market.

## Materials and methods

**Fig. 1:**
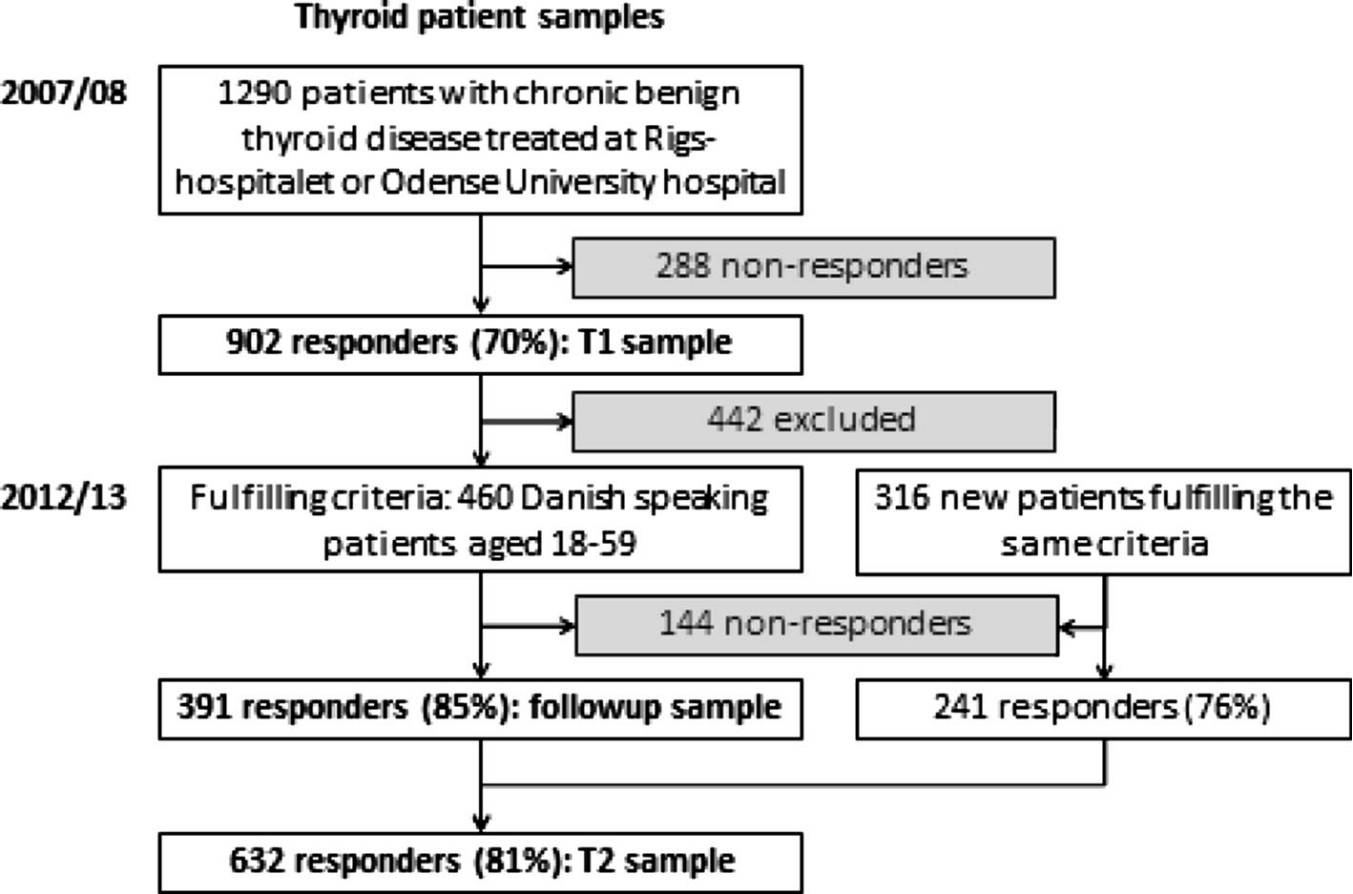
Flow chart of the thyroid patient population

This study is an extension of the ThyPRO validation study [39]. In 2007/2008 (time 1), patients were recruited from the endocrine outpatient clinics of two Danish hospitals: Copenhagen University Hospital, Rigshospitalet (RH), and Odense University Hospital (OUH). Patients were included if they had one of the following diagnoses: non-toxic goiter, toxic nodular goiter, Graves’ disease (with or without orbitopathy), autoimmune hypothyroidism, and other thyroid diseases (for example, postpartum thyroiditis and subacute thyroiditis) and were between 18 and 59 years. Exclusion criteria were as follows: Serious comorbidity (e.g., cancer) and inability to complete a questionnaire due to language problems (non-Danish speaking, blindness, etc.). Out of 1,290 patients, 902 returned the ThyPRO questionnaire (Fig. 1).

In 2012/2013 (time 2), 460 patients from the time 1 sample were invited for the work ability study if they were between 18 and 59 years. The other 442 patients were excluded for the following reasons: outside the age range (323), unidentifiable addresses or declined (74), died or emigrated (41), and wrong diagnosis upon re-examination (4). In addition, 316 new patients were recruited from the same hospitals, using the same inclusion and exclusion criteria. The work ability questionnaire was sent to the combined sample of 776 patients in the period May 1, 2012–May 1, 2013. In case of non-response, reminders were sent after 2 and 4 weeks. After 5 weeks, Statistics Denmark contacted all non-responders by phone. Of the total sample 632 responded (time two sample—81 %), 391 of which had also participated at time 1 (the follow-up sample, see Fig. 1).

At time 2, responders were significantly older (mean age 46 years) than non-responders (mean age 42 years, *p* < 0.0001) and significantly more likely to be employed (80 % compared to 70 %, *p* < 0.01). No differences were found regarding job type, work sector, type of diagnosis, or years from diagnosis.

### Control population

Data from the general Danish population stemmed from the National Work and Health study conducted in 2012 (NWHS 2012) [40]. Participants answered three items on work ability, which were also used in the time 2 study. We excluded participants who were above 59 years (*n* = 1,358) or had more than one missing value in one of the three work ability questionnaire items applied in this study (*n* = 979), leaving a total sample of *n* = 15,050 for this study.

### Development of the work ability questionnaire

We selected, revised, and developed items based on the literature, review of work ability questionnaires, and interviews with thyroid patients [32]. By literature review, we identified the work ability constructs, including self-report items that measure the ability to carry out activities while at work. We did not include items that entail socioeconomic aspects, safety issues, or accidents at work. We prioritized inclusion of items that were already developed and validated. However, in order to cover all the specific themes of importance to patients with thyroid disease, we also developed new items. All were evaluated by a panel of experts within social science or endocrinology. In order to evaluate whether the items were perceived as intended, the questionnaire was tested and revised through cognitive interviews with 40 patients at OUH and RH.

### Work ability constructs and items

**Fig. 2:**
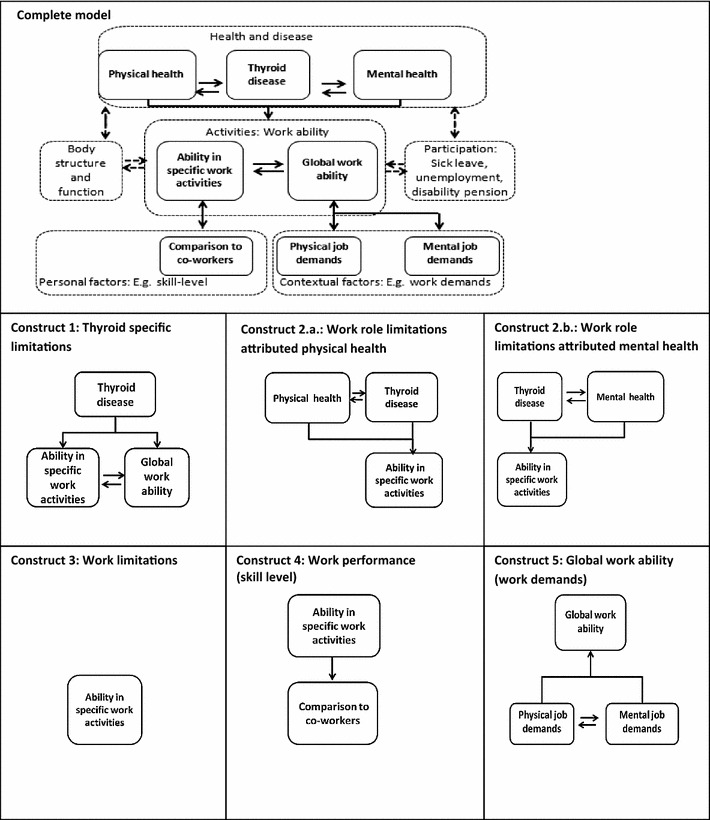
A model for work ability, and five strategies for assessment of work ability

Five different work ability constructs (Fig. 2) with a total of 24 self-report work ability items were included in the questionnaire (Table 1):

**Table 1:** Overview of work ability items administered at time 2

Constructs (abbreviation)	Work ability questions, [item abbreviation], (response scale), source of item/scale
1. Thyroid-specific limitations (THY)	During the past 4 weeks, has your thyroid disease caused you to have difficulty managing your job (for example, had difficulty managing it or calling in sick)? [T_DIFF]^a^
	(Not at all, a little, some, quite a bit, very much) 1 item from the ThyPRO questionnaire
	During the past 4 weeks, has your thyroid disease caused you to
	Take off from work earlier than usual? **[**T_OFF]
	Plan your work differently [T_PLAN]
	Involved your employer or colleagues in order to manage your work? [T_INVOLVE]
	(To a very little extent, to a little extent, somewhat, to a high extent, to a very high extent)
	Newly developed items
2.a. Work role limitations attributed to physical health (RP)	During the past 4 weeks, have you had any of the following problems with your work or other regular daily activities as a result of your physical health?
	Cut down on the amount of time you spent on work or other activities? [SF36_RP1]
	Accomplished less than you would like [SF36_RP2]
	Were limited in the kind of work or other activities? [SF36_RP3]
	Had difficulty performing the work or other activities (for example, it took extra effort)? [SF36_RP4]
	Four items from the SF-36-v2 role physical scale
2.b. Work role limitations attributed to mental health (RE)	During the past 4 weeks, have you had any of the following problems with your work or other regular daily activities as a result of any emotional problems (such as feeling depressed or anxious)?
	Cut down on the amount of time you spent on work or other activities? [SF36_RE1]
	Accomplished less than you would like? [SF36_RE2]
	Did your work less carefully than usual? [SF36_RE3]
	(All of the time, most of the time, some of the time, a little of the time, none of the time)
	Three items from the SF-36-v2 role emotional scale
3. Work Limitations (WL)	In the past 2 weeks, did you have difficulties with
	Working the required amount of hours? [WL_HOURS]
	Doing your work without taking extra breaks? [WL_BREAK]
	Stick to a routine or a plan? [WL_ROUTINE]
	Control your temper when working with others? [WL_TEMPER]
	Keep your mind on your work tasks? [WL_ATT]
	(All of the time, most of the time, some of the time, a little of the time, none of the time)
	Five newly developed items
4. Work performance (PERF)	Comparing yourself to others, who have the same type of work, how do you estimate your own ability to
	Handle a big work load? [P_LOAD]
	Do your job well? [P_WELL]
	Work without making mistakes? [P_ERROR]
	Make quick decisions? [P_DECIDE]
	Concentrate on your work? [P_CONC]
	(A lot better, a little better, about the same, a little worse, a lot worse)
	Complete work performance scale from the Danish National Working Environment Survey
5. Global work ability (Global)	Assume that your work ability at its best has a value of 10 points. How many points would you give your current work ability? [G_WAI]^b^
	(11 point scale from 0 to 10; 0 = currently not able to work at all, 10 = work ability at its best)
	How do you rate your current work ability with respect to
	The physical demands of your work? [G_PHYS]^b^
	The mental demands of your work? [G_PSY]^b^
	(Excellent, very good, good, fair, poor)
	Three items from the Work Ability Index

^a^Item also administered at time 1

^b^Item also administered to the general population

1. *Thyroid-specific limitations* assess limitations in work activities attributed to the thyroid disease. One item from the THYPRO [39] assessed the impact of thyroid disease on work performance in general. Data on this item were available at time 1 and 2. We developed three new items on the impact of thyroid disease on specific work aspects and identified in the previous qualitative study [32] (Table 1).
2. *Work role limitations attributed to physical (2.a.) or mental (2.b.)* health concerns limitations in work or daily role function attributed to either physical or emotional problems. We selected seven items from the SF-36v2 [38, 41–44].
3. *Work limitations* include five newly developed items that addressed the difficulties in the ability to perform specific work activities. Previous studies [17, 31, 32] identified activities posing emotional and cognitive demands as particularly challenging for thyroid patients. We initially selected five items from the WLQ [37] that covered this content. However, since cognitive interviews identified problems in item interpretation, we revised the items to simplify the questions and response categories. The final items did not use attribution to health, individual, or contextual factors.
4. *Work performance* refers to the employees’ experienced ability to perform at work compared to the ability of co-workers. These five items were included because they consider the skill level in the conceptualization of work ability. We used items adapted from the Work Performance Questionnaire [45] previously applied in the Danish National Working Environment Survey [46] and The Danish Work Environment Cohort Study (2010) [47, 48].
5. *Global work ability* refers to a person’s global assessment of his or her ability to work. We included three items from the WAI [36]: One item measured global work ability compared to the best ever, and two items also considering the mental and physical demands of the job. WAI has shown validity in working populations [49] and among clinical populations [50, 51]. The items have been included in National Danish Surveys [40, 47, 48] allowing us to compare the responses of the thyroid patients to the responses from the general population.

### Clinical measurements

Date of diagnosis was obtained by chart review by medical staff. An endocrinologist classified patients by their initial diagnosis. There were six diagnostic categories: (1) non-toxic goiter (diffuse, uninodular and multi-nodular non-toxic goiter and thyroid cysts), (2) toxic nodular goiter (uninodular and multi-nodular toxic goiters), (3) Graves’ disease, (4) Graves’ orbitopathy (GO), (5) autoimmune hypothyroidism, and (6) other thyroid diseases (postpartum and subacute thyroiditis).

### Covariates

Information on gender, age, job type, and work sector was identified via the central population register (CPR register) at Statistics Denmark. Age was defined as age in years at the time of response to the survey or set for non-responders at January 1, 2013. Job type was classified via the Danish version of the International Standard of Classification of Occupations (DISCO-08) [52, 53] and aggregated into three categories according to high, medium, and low cognitive job demands. Work sector was classified by the European Classification of Economic Activities [54] and aggregated into three main categories: (1) production and trade, (2) finance and real estate, and (3) knowledge, health, and public administration sectors.

### Statistical analyses

Aim 1 was pursued through factors analysis and tests of differential item functioning (DIF). The factor structure of all of the 24 work ability items was evaluated through confirmatory factor analyses (CFA), evaluating three models: (1) a unidimensional model where all items loaded on one factor, (2) a bi-factor model [55] where all items loaded on a global factor, and items additionally loaded on sub-factors defined by item content (thyroid-specific limitations, work role limitations attributed physical or mental health, work limitations, and work performance). The bi-factor model was revised until a satisfactory fit was achieved, and (3) a multifactor model, specifying the sub-factors identified above as correlated factors and dropping the global factor. All items were considered effect indicators of the latent factors (reflective model). The factor analyses were conducted using the MPlus software and polychoric correlations using weighted least-squares parameter estimation with mean and variance adjustment (WLSMV) [56]. Models were evaluated using the comparative fit index (CFI) >0.95 [57] and the root mean square error of approximation (RMSEA) <0.08 [57] as criteria for acceptable model fit. Finally, residual correlations were examined to evaluate the local independence of items.

DIF in relation to age, gender, and thyroid disease was evaluated with ordinal logistic regression [58]. DIF is seen if an item has a unique interpretation for persons in a particular subgroup or if an item has an association with group membership that differs from the other items in the particular scale. Maximum likelihood estimation with the Newton–Raphson method was used in SAS (version 9.3). The extent of DIF was described by the pseudo *R* ^2^ statistics as defined by Nagelkerke [59], and ∆*R* ^2^ ≥ 0.02 was defined as notable DIF [60].

Aims 2 and 3 were evaluated by head-to-head comparisons of the single items and scales to identify the measures with highest relative validity [61]. Aim 2 and hypothesis a were evaluated by comparing the responses of people with six different thyroid diseases on the three single items from the WAI (G_WAI, G_PHYS, G_PSY) to the responses from the general population using ordinal logistic regression.

Aim 3 and hypothesis b were evaluated by comparing the responses of six different thyroid disease groups (reference group = non-toxic goiter) on the work ability scales identified in the CFA using linear regression analysis. Scale scores were calculated as the mean of the item scores and transformed to a metric from 0 (worst) to 100 (best work ability). We also compared patients diagnosed within the previous year to patients diagnosed more than a year (hypothesis c). The analyses (aim 2 and 3) were adjusted for age, gender, job type, and work sector.

Aim 4 was evaluated using participants who were employed and answered the work ability item at time 1 and were reassessed at time 2. Using logistic regression, we estimated the odds ratios (OR) of being excluded from the labor market at time 2, if reporting work disability (‘a little’, ‘some’, ‘quite a bit’, or ‘very much’) at time 1 on a single item of thyroid-specific work ability (THY_DIF, Table 1). Participants were regarded as excluded from the labor market at time 2 if they were unemployed or received disability pension. Patients who were unemployment or received disability pension were identified via registers of labor market statistics and CPR register at Statistics Denmark. We adjusted for age, gender, and education.

Except the CFA analysis, all analyses were performed with SAS (version 9.3).

## Results

Compared with the general population, the clinical population included more subjects above the age of 29 years and more women (Table 2). A larger percentage of thyroid patients worked in the knowledge and health sectors, and a larger percentage had jobs with high cognitive demands.

**Table 2:** Characteristics of the thyroid patient sample at time 2 and of the general population sample

	Thyroid patients		General population
	All responders (*n* = 632) (%)	Employed (*n* = 507) (%)	Employed (*n* = 15,050) (%)
Diagnoses			
Non-toxic goiter	30	37	
Toxic nodular goiter	2	9	
Graves’ disease	20	20	
Graves’ Orbitopathy	7	6	
Autoimmune thyroid hypothyroidism	23	23	
Other thyroid diseases	10	5	
Missing	<1	<1	
Years with diagnosis			
6 or more years	39	38	
2–5 years	28	30	
0–1 years	31	30	
Missing	2	2	
Age*			
18–29	5	3	12
30–39	19	19	20
40–49	37	39	33
50–59	39	39	35
Females*			
Females	88	87	45
Cognitive job demands*			
High	33	40	35
Medium	10	11	13
Low	33	38	45
Missing	25	11	7
Work sector*			
Production	7	8	23
Finance	25	28	26
Knowledge and health	47	57	36
Missing	21	7	15

* Significant difference between employed thyroid patients and the general (Chi-square test)

### Results from CFA and DIF

The results of the CFA are presented in Table 3. In a one-factor model, all items had factor loading >0.60, except for one item (WL_TEMPER) that had a loading of 0.52. However, model fit was poor (CFI = 0.79 and RMSEA = 0.26). A bi-factor model with five sub-factors achieved a satisfactory fit after allowing residual correlations between three items, which all contained the phrase “work ability,” and two items about working well and working without errors (P_WELL, P_ERROR). While most items had strong loadings on the global factor, many items also had high loadings on the specified sub-factors. Higher loading on a sub-factor than on the general factor was only seen for items in the performance scale. However, fairly high loadings on sub-factors were also seen for items on work role limitations due to emotional problems, items with specific reference to thyroid disease, and items (without disease attribution) on work limitations in specific work areas. The items with highest loadings on the global factor was a general item on work ability (G_WAI), an item on difficulties in doing the job due to thyroid disease (THY_DIF), items on role limitations due to physical disease, and an item on working the required number of hours (WL_HOURS).

**Table 3:**
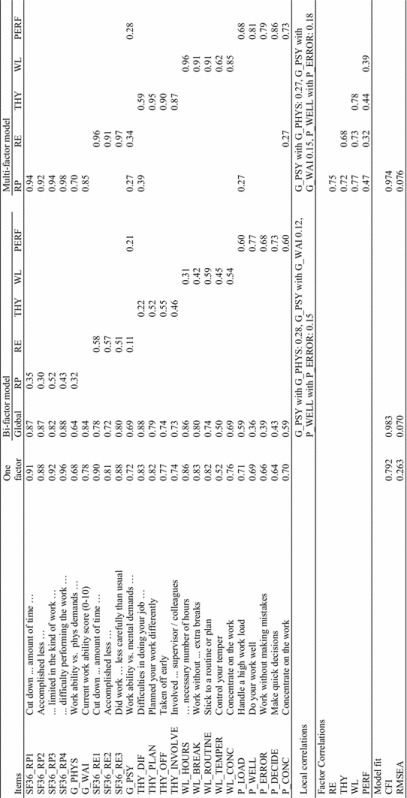
Factor analysis of work ability items in thyroid patients at time 2

*RP* role physical, *RE* role emotional, *THY* thyroid-specific limitations, *WL* work limitations, *PERF* work performance, *CFI* comparative fit index, *RMSEA* root mean square error of approximation

A multifactor model with five correlated factors also attained acceptable fit after allowing the same residual correlations as in the previous model. The factor structure resembled the factors identified in the bi-factor model with a few notable exceptions: The global item on work ability (G_WAI) and the global item on work ability in relation to physical work demands (G_PHYS) loaded clearly together with the SF-36 items on role limitations attributed to physical health. An item on WA in relation to mental work demands (G_PSY) loaded on several factors, but did not load strongly on any one factor. While a few cross-loadings were found for the remaining items, the factor pattern was clear. Thus, the five factors identified in the analysis were as follows: (1) work role limitation, including the three SF-36 Role Physical (RP) items and two global WA items, (2) work role limitations due to mental health: role emotional (RE), (3) work limitations attributed to thyroid disease (THY), (4) a factor on work limitations (WL) without health attribution, and (5) a work performance (PERF) factor on WA as compared to co-workers.

We pursued the scales based on this factor structure with one exception. Although the G_WAI and G_PHYS items loaded strongly together with the SF-36 RP items, we decided not to score them as part of the role physical scale, but regarded them as independent constructs as illustrated in Fig. 2.

Results from the DIF analyses showed that two items in the work limitations scale showed either uniform DIF (WL_ROUTINE) or non-uniform DIF (WL_TEMPER) with regard to age. These items were removed from the WL scale.

### Results from general population comparison

In comparisons between thyroid patients and the general population, the global item on work ability (G_WAI) had higher validity than items considering the physical or mental demands of the work (Table 4). Hypothesis a was only partly confirmed: Graves’ disease, autoimmune hypothyroidism, and other thyroid diseases, but not people with goiters or Graves’ orbitopathy, had significantly lower scores compared to the general population on the global work ability item (G_WAI). Patients with Graves’ disease also rated their work ability worse with respect to mental demands. Patients with non-toxic goiter rated their work ability better than the general population on the two items on work ability in relation to physical and mental demands.

**Table 4:** Work ability (WA) compared to the general population at time 2

Effect^a^	Global work ability (WA)		WA with respect to mental demands		WA with respect to physical demands	
	OR	95 % CI	OR	95 % CI	OR	95 % CI
Non-toxic goiter (*n* = 186)	1.11	(0.84–1.48)	**0**.**71**	(**0**.**53**–**0**.**95**)	**0**.**73**	(**0**.**54**–**0**.**98**)
Toxic nodular goiter (*n* = 45)	0.93	(0.52–1.66)	0.90	(0.50–1.63)	0.84	(0.47–1.53)
Graves’ disease (*n* = 99)	**3**.**14**	(**2**.**16**–**4**.**57**)	**1**.**79**	(**1**.**22**–**2**.**63**)	1.44	(0.98–2.12)
Graves’ orbitopathy (*n* = 32)	1.79	(0.92–3.51)	0.67	(0.34–1.35)	0.66	(0.32–1.35)
Autoimmune hypothyroidism (*n* = 115)	**1**.**68**	(**1**.**18**–**2**.**4**)	0.83	(0.58–1.19)	0.71	(0.49–1.03)
Other thyroid disease (*n* = 26)	**2**.**12**	(**1**.**06**–**4**.**26**)	0.66	(0.32–1.36)	1.24	(0.60–2.56)
Chi-square (RV) 6 *df*	**50**.**8**	(**1**)	**18**.**8**	(**0**.**37**)	**13**.**2**	(**0**.**26**)

Odds ratio (OR) for a one category LOWER score with 95 % confidence intervals (CIs)

Significant differences in bold

*RV* relative validity

^a^Compared to the general population (*n* = 15,408). Estimates adjusted for gender, age, job type, and work sector

### Results from work ability scales comparisons

In head-to-head comparisons, the statistical strength varied considerably between the five scales. The scale with thyroid disease attribution (THY) provided the most power in the comparison of disease subgroups (*F* = 5.9, 5 *df*, Table 5) and with regard to disease duration (*F* = 12.1, 1 *df*). The RP and RE scales from the SF-36 also showed significant differences between disease subgroups and with regard to disease duration.

**Table 5:** Analysis of work ability for thyroid disease subgroups at time 2

Parameter	Work role physical		Work role mental		Thyroid-specific limitations		Work limitations		Work performance	
	Est.	95 % CI	Est.	95 % CI	Est.	95 % CI	Est.	95 % CI	Es.t	95 % CI
Reference group score^a^	52.8	(50.8/54.8)	52.7	(50.6/54.9)	99.4	(95.7/103.1)	87.7	(82.2/93.3)	56.8	(52.8/60.8)
Differences from the reference group										
Toxic nodular goiter (*n* = 45)	−1.8	(−4.8/1.2)	−1.8	(−5.1/1.4)	−5.1	(−10.8/0.5)	−2.7	(−11.1/5.7)	−1.2	(−7.2/4.9)
Graves’ disease (*n* = 99)	**−4**.**0**	(**−6**.**3/−** **1**.**7**)	**−2**.**8**	(**−5**.**2/−** **0**.**4**)	**−11**.**4**	(**−15**.**6/−** **7**.**2**)	**−9**.**4**	(**−15**.**7/−** **3**)	−4.2	(−8.7/0.4)
Graves’ orbitopathy (*n* = 32)	−1.2	(−4.7/2.2)	−3.5	(−7.2/0.1)	−5.2	(−11.6/1.2)	−2.7	(−12.5/7)	3.6	(−3.4/10.6)
Autoimmune hypo. (*n* = 115)	−2.1	(−4.2/0.1)	**−3**.**4**	(**−5**.**7/−** **1**.**2**)	**−4**.**2**	(**−8**.**2/−** **0**.**2**)	−4.5	(−10.5/1.5)	−1.0	(−5.3/3.3)
Other thyroid d. (*n* = 26)	−1.9	(−5.4/1.7)	−1.2	(−4.9/2.6)	−1.4	(−7.9/5.2)	−5.8	(−15.6/4)	−3.5	(−10.5/3.6)
Disease <1 year^b^	**−2**.**6**	(**−4**.**4/−** **0**.**8**)	**−2**.**2**	(**−4**.**1/−** **0**.**3**)	**−5**.**9**	(**−9**.**3/−** **2**.**6**)	−4.2	(−9.2/0.8)	**−3**.**8**	(**−7**.**4/−** **0**.**2**)
*F* value (RV) 5 *df*	**2**.**5**	(**0**.**42**)	**2**.**3**	(**0**.**39**)	**5**.**9**	(**1**)	1.7	(0.29)	1.2	(0.20)
*F* value (RV) 1 *df*	**8**.**3**	(**0**.**69**)	**5**.**4**	(**0**.**45**)	**12**.**1**	(**1**)	2.7	(0.22)	**4**.**2**	(**0**.**35**)

Significant differences in bold

*RV* relative validity

^a^Reference group (ref.) = patients with non-toxic goiter (*n* = 223). Estimates adjusted for gender (ref. = female), age (ref. = over 45 years), job type (ref. = job with low cognitive demands), and work sector (ref. = the knowledge and health sector)

^b^Ref. = disease for more than 1 year

Hypothesis b was partly confirmed, as significant impact of four out of five scales (RE, RP, THY, WL) was found in patients with Graves’ disease, and of two scales (RE, THYR) in patients with autoimmune hypothyroidism (Table 5). Score differences for the other clinical subgroups did not achieve statistical significance. In line with hypothesis c, patients rated their work ability worse within the first year of diagnosis.

### Results from analysis of predictive validity

The single item (THY_DIFF) from the THYR scale showed good predictive validity. Participants who reported thyroid associated work limitations at time 1 were 5 times more likely to be excluded from the labor market at time 2 (OR 5.0, 95 % CI 2.7–9.1 adjusting for age, gender, and education).

## Discussion

Although almost all the work ability items had strong loadings on one factor, we identified the multifactor model as the best model for two reasons. Loadings on sub-factors were of sufficient magnitude to empirically justify this model, and this model was also in better accordance with our original theoretical assumptions (Fig. 2). We found only a few instances of DIF, which were solved by deleting the items in question (aim 1).

The results from CFA showed that the five-factor model deviated from our theoretical assumptions (Fig. 2) in one respect: WAI items (global work ability) and work role items with attribution to physical health (SF-36) loaded on the same factor. Since these items both focus on physical aspects of work ability, they have similar content. However, the items derive from two different constructs that fundamentally differ with regard to specificity of the question and the way they assess the impact of health on work ability: Items from the WAI assess overall work ability in relation to physical work demands, but the SF-36 assess difficulties with performing specific activities at work or in daily life with attribution to physical health [35, 62]. Consequently, we decided to maintain our original theoretical distinction between these constructs.

In head-to-head comparisons of the three global work ability items (aim 2), the simple global item (G_WAI) was most effective in discriminating between thyroid patient subgroups and the general population. This single item has also been identified as a strong predictor of sickness absence and early retirement [63]. However, the other two items requiring direct assessment of physical and mental work demands discriminated less well and also lead to the non-intuitive result that patients with non-toxic goiter had better work ability than the general population. Previous research has shown better discriminate validity of G_WAI over the entire index [62] and has shown that this single item is easier to understand than questions requiring assessment of mental or physical work demands [64]. It is also possible that assessment of ability in relation to work demands triggers a social desirability effect in this particular clinical population that explains the non-intuitive results of the present study.

Previous literature found that disease-specific QOL measures provided more statistical power than generic items [65, 66], but a similar comparison has not been made in relation to work ability measurements. In head-to-head comparisons of the five work ability scales (aim 3), the thyroid-specific scale provided the most power for discriminating between the diseases, followed by the SF-36 work role functioning scales. The work limitations scale discriminated less well and the work performance scale failed to discriminate at all, suggesting that questions that include attribution to health or disease are better than questions with no attribution at all. The work performance scale required assessment of individual skill level (comparison of ability to co-workers), which may contribute to the poor discrimination ability of this scale.

Hypothesis c) was supported, but (a) and (b) were only partly supported. Low self-assessed work ability was particularly seen for patients with Graves’ disease and autoimmune hypothyroidism. The autoimmune component is characteristic for both diseases, and the findings are in line with previous studies, suggesting that the autoimmune component of hypothyroidism and hyperthyroidism, as opposed to thyroid dysfunction per se, may be associated with more serious disability [67, 68]. However, since relatively few patients were included in some of the thyroid subgroups, this question requires further study. Previous studies [25, 26] have found a significant impact of Graves’ orbitopathy on work ability. It is possible that our nonsignificant results may be due to early retirement of the most severely affected patients (similar to the healthy worker effect) [69].

An item from the thyroid-specific scale showed good predictive validity as it predicted early involuntary retirement (aim 4). This item assessed the experienced difficulties managing the job, and the results suggest that this item was a valid indicator of the long-term socioeconomic consequences of having a thyroid disease.

## Conclusion

Although the different work ability constructs were related, they could not be seen as one general construct (aim 1). Of the five identified work ability scales, the scale on work limitations with attribution to thyroid disease was most effective in detecting impact on work ability for people with thyroid diseases (aim 3) and predicting exclusion from the labor market (aim 4). For comparisons with the general population or other disease groups, the role functioning scales from the SF-36 and/or the single global item from the WAI appear useful (aim 2). These scales and this item can also be used with patients that are out of work.
